# Ultrastructure of Placenta of Gravidas with Gestational Diabetes Mellitus

**DOI:** 10.1155/2015/283124

**Published:** 2015-08-24

**Authors:** Qian Meng, Li Shao, Xiucui Luo, Yingping Mu, Wen Xu, Chao Gao, Li Gao, Jiayin Liu, Yugui Cui

**Affiliations:** ^1^Department of Obstetrics, Lianyungang Maternity and Child Health Care Hospital, Lianyungang 222000, China; ^2^The State Key Laboratory of Reproductive Medicine, Center of Clinical Reproductive Medicine, First Affiliated Hospital of Nanjing Medical University, 300 Guangzhou Road, Nanjing 210029, China

## Abstract

*Objectives*. Gestational diabetes mellitus (GDM) leads to an abnormal placental environment which may cause some structural alterations of placenta and affect placental development and function. In this study, the ultrastructural appearances of term placentas from women with GDM and normal pregnancy were meticulously compared. *Materials and Methods*. The placenta tissues of term birth from 10 women with GDM and 10 women with normal pregnancy were applied with the signed informed consent. The morphology of fetomaternal interface of placenta was examined using light microscopy (LM) and transmission electron microscopy (TEM). *Results*. On LM, the following morphological changes in villous tissues were found in the GDM placentas when compared with the control placentas: edematous stroma, apparent increase in the number of syncytial knots, and perivillous fibrin deposition. On TEM, the distinct ultrastructural alterations indicating the degeneration of terminal villi were found in the GDM placentas as follows: thickening of the basal membrane (BM) of vasculosyncytial membrane (VSM) and the VSM itself, significantly fewer or even absent syncytiotrophoblastic microvilli, swollen or completely destroyed mitochondria and endoplasmic reticulum, and syncytiotrophoblasts with multiple vacuoles. *Conclusion*. Ultrastructural differences exist between GDM and control placentas. The differences of placenta ultrastructure are likely responsible for the impairment of placental barrier and function in GDM.

## 1. Introduction

Gestational diabetes mellitus (GDM) is defined as the glucose intolerance with onset or first recognition during pregnancy [1]. GDM affects 4%–7% of pregnant population in the United States [2, 3] and 6.8% in China [4]. GDM is associated with short- and long-term morbidity in both offspring and mother. The short-term adverse outcomes include macrosomia, neonatal hypoglycemia, neonatal jaundice, preeclampsia, preterm delivery, and cesarean delivery, while the long-term complications include obesity, abnormal glucose tolerance and diabetes in adolescence, or early adulthood. Meanwhile, the concept of the fetal origin diseases has been seriously taken including imprinting genes and epigenetics study.

Placenta plays critical roles during pregnancy, including the exchange of nutrients, water, respiratory gases, and waste products and the synthesis of various hormones which regulate the transport of maternal fuels to the fetus and facilitate maternal metabolic adaptation to different pregnancy stages. These functions are determined by the structure, ultrastructure, and function of the placental exchange barrier. The vasculosyncytial membrane (VSM) [5, 6] is an important feature of placental barrier, which is composed of different overlapping structures including a continuous maternal-facing syncytiotrophoblastic layer with multiple apical microvilli, the endothelial lining and underlying basal membrane (BM) of the fetal capillaries, and the villous connective tissue between them. The most significant properties of VSM are to maintain the exchange surface area between the fetomaternal surfaces [7]. The increased thickness of VSM and reduced area of exchange related to fetal hypoxia appear to subject fetus to considerable risks [8].

It was found that several ST cells in GDM placentas showed the dilation of rough and smooth endoplasmic reticulum (ER), loss and alteration of microvilli, large vacuoles just beneath the plasma membrane, and mitochondrial irregularities [9]. However, since the placenta is a structurally complex organ composed of many cell types with different origins, it is necessary to obtain the detailed information using systematic ultrastructural examinations. In this study, the morphological and ultrastructural appearances in GDM placentas were investigated using both light microscopy (LM) and transmission electron microscopy (TEM), so as to explore its effect on the placenta functions and fetal development, as well as the fetal origin diseases.

## 2. Materials and Methods

### 2.1. Subjects

A 75-goral glucose tolerance test (OGTT) was performed with plasma glucose measurement fasting and at 1 and 2 h for women at 24–28 weeks of gestation who were not previously diagnosed with overt diabetes. The OGTT should be performed in the morning after an overnight fast of at least 8 h. The diagnosis of GDM is made when any of the following plasma glucose values are exceeded based on the American Diabetes Association (2011) [1]: fasting *⩾*5.1 mmol/L (92 mg/dL); 1 hour *⩾*10.0 mmol/L (180 mg/dL); 2 hours *⩾*8.5 mmol/L (153 mg/dL). Women with a history of pregestational diabetes and those with a nonsingleton index pregnancy were excluded. After GDM was diagnosed, those GDM women were asked to control diet in order that their fasting blood glucoses were kept in the satisfying range (3.3 to 5.6 mmol/L). Until delivery, eight GDM women (8/10) kept in the A1 class (fasting glucose less than 5.8 mmol/L, postprandial blood glucose less than 6.7 mmol/L) after good diet control (see Supplemental Table 1 in Supplementary Material available online at http://dx.doi.org/10.1155/2015/283124). Two GDM women (2/10) were classed as A2 (fasting blood glucose higher than or equal to 5.8 mmol/L, postprandial blood glucose higher than or equal to 6.7 mmol/L) because of their poor diet control. The levels of fasting blood glucose and HbA1c in those GDM women with good diet control in the last weeks of gestation were kept in the range of 5.1 to 5.6 mmol/L. However, level of fasting blood glucose (6.1 and 11.3 mmol/L) and level of HbA1c (10.2 and 10.3 mmol/L) in two GDM women with poor diet control were higher than the satisfactory criteria. The clinical data on maternal age and weight, number of gestational weeks, mode of delivery, birth weight, and weight of placenta were summarized in Table 1. Women with normal pregnancies matched with GDM women for number of gestational weeks, maternal age, and mode of delivery were recruited as control (Supplemental Table 2). In each group, 10 placental samples were collected with the signed infoemed consent. The study procedure was approved by the Ethics Committee of the First Affiliated Hospital of Nanjing Medical University.

### 2.2. Placental Sample Collection

All selected subjects adopted the cesarean delivery due to other reasons. Placental specimens were weighed and performed on the basis of obstetric indications. The issue specimen was dissected from the placental subchorial zone corresponding to the umbilical cord insertion (~5 cm away from the site of cord insertion), while avoiding area of infarction and hematomas. Placenta with abnormal umbilical cord insertions, such as velamentous cord insertion, was excluded. Fragments from the placenta were cut longitudinally from the maternal side to the fetal side. Placental tissues were divided into three parts, as described by Sood et al. [10]: maternal, middle, and fetal. The middle part, comprising homogeneous villous tissues, was collected and floated in ice-cold phosphate-buffered saline (PBS), cleaned of blood, and immediately cut into double four 1 × 1 × 1 cm fragments, which were fixed either with 4% paraformaldehyde or with 2.5% glutaraldehyde at 4°C for further LM and TEM examinations, respectively. To minimize the variation among villous tissue blocks collected from four different placental sites in both groups, four blocks from each placenta were randomly examined.

### 2.3. LM Examination

Three paraffin-embedded blocks from four paraformaldehyde-fixed tissues and from each placenta were randomly selected. Sections (thickness, 4 *μ*m) were cut from each block and stained with hematoxylin and eosin (HE). The slides were observed under an Axioskop 2 Plus microscope (Carl Zeiss) and photographed. The HE-stained sections were carefully analyzed to provide a general view of the section and to confirm that the sections had appropriate histological features and were suitable for the subsequent TEM examination.

### 2.4. TEM Examination

From each placenta, three villous tissue blocks were prepared for ultrastructural examination using TEM. The samples were fixed in 2.5% glutaraldehyde in cacodylate buffer and stored in cacodylate buffer with 0.05 M saccharose (pH, 7.2) at 4°C until processing. The villous tissues were subsequently postfixed in 1% OsO_4_ for 2 h at 4°C, routinely processed in a graded series of acetone, infiltrated with acetone-araldite, and embedded in araldite. For orientation, semithin sections (thickness, 1 *μ*m) were stained with thionine. Ultrathin sections (thickness, 80 nm) were treated (double contrast) with uranyl acetate (25 min) and 8% lead nitrate (5 min) and then systematically examined using a JEM-1010 electron microscope (JEOL Ltd.).

Terminal villi were evaluated with respect to the placental blood barrier (thickness of VSM, thickness of syncytiotrophoblast BM, and thickness of endothelial BM), villous stroma (villous edema), and cytotrophoblasts (CTs) and syncytiotrophoblast (ST) along with their substructures (microvillous density per unit surface area, cytoplasmic vacuolization, pyknosis, mitochondria, and ER). Ultrastructural findings were compared with quantitative measured data and assigned a semiquantitative score based on the degree of alteration in the ultrastructure (Table 2). Five fields of vision were randomly selected from each tissue block and systematically investigated at 5,000x, 12,000x, and 25,000x magnification for quantitative analysis. Thus, 15 random fields of vision were recorded and analyzed per placenta to minimize interindividual differences. In each field of vision, the following three measurements were performed: (1) thickness of VSM was measured from the intervillous space to the fetal vessels, perpendicular to the BM, under 5,000x magnification, (2) microvillous density was counted per 10 *μ*m of length under 12,000x magnification, and (3) thickness of the ST BM and endothelial BM was measured under 25,000x magnification. Images were analyzed using the TEM Image Platform (Olympus) to perform random measurements. Two operators severally performed the microscopic analyses and were blinded to the placental groups. The third person collected data from two operators and performed statistical analyses.

### 2.5. Statistical Analysis

Data were expressed as the mean and standard deviation (SD). Statistical analyses were performed using the SPSS software (Statistical Analysis System, version 17.0 for Windows). Statistical difference between two groups was analyzed using the Student *t*-test. Statistical significance was set at *P* < 0.05.

## 3. Results

### 3.1. Histological Examination

**Figure 1:**
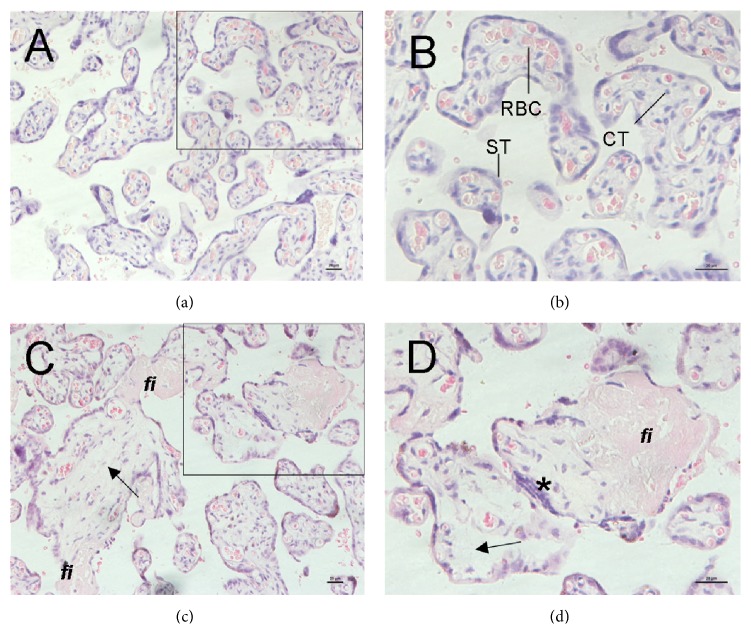
Pathological morphology of placentas from control group (a, b) and GDM group (c, d). Placental sections were HE-stained. (a) The normal branching of villous trees. (b) The syncytiotrophoblast (ST) was clearly observed lying outside the villi, with scarce single cells of cytotrophoblast (CT). (c and d) The increased syncytial knotting (*∗*), villous edema (arrows), and perivillous fibrin (fi) depositions in GDM group. Bar = 20 um.

The normal morphophysiological characteristics of term placenta of control group were showed in Figures 1(a) and 1(b). Multigrade branching of villous trees lined with STs was observed. The fetal blood vessels were remarkable with clearly evident erythrocytes inside multiple placental villi. No obvious calcification processes, fibrin deposition, or villous edema was found. Typical morphological figures of term placenta of GDM group were showed in Figures 1(c) and 1(d). In some area of slides of GDM placentas, the following morphological changes in terminal villi were qualitatively showed: edematous stroma, apparent increase in the number of syncytial knots, and perivillous fibrin deposition.

### 3.2. Ultrastructural Examination

**Figure 2:**
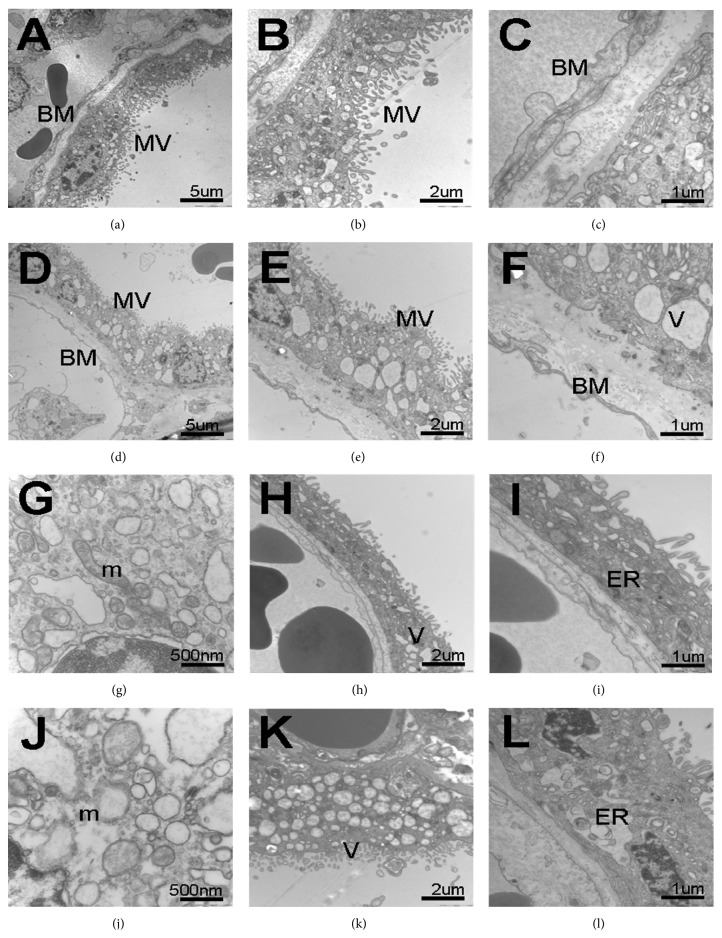
Ultrastructure of placentas from control group (a–c, g–i) and GDM group (d–f, j–l) observed using a JEM-1010 electron microscope. TEM images showed at different magnifications (×5000 (a and d), ×12 000 (b and e), and ×25 000 (c and f)). (a–c) Normal placental barriers were displayed in control, VSM, composed of the outer layer ST with a multitude of microvilli (MV) on the surface, fetal capillaries with fetal blood cells inside, and BM and interspaces between them. (d–f) In GDM group, intact placental barriers were apparent along with degenerative alterations of the terminal villi, mainly in VSM and BM, including thicker placental barriers, decreased apical microvilli, and increased multiple vacuoles (V) in ST. Bar = 5 um (a and d), 2 um (b and e), and 1 um (c and f). (g–i) Mitochondria (g), cytoplasmic vacuoles (h), and ER (i) with normal appearance in the cytoplasm of ST in control group. (j) The massive swelling, even ridge deprivation, and architectural disruption of mitochondria (m) in the GDM placenta. (k) The massive cytoplasmic vacuoles accumulation in the ST of GDM placenta. (l) The dilation of endoplasmic reticulum (ER) cisternae in GDM group. Bar = 500 nm (g and j), 2 um (h and k), and 1 um (i and l).

In the control group, the major structure of placental barrier included the STs, endothelium and their underlying BMs were clearly showed in Figures 2(a) to 2(c). The STs were arranged in a monolayer, with multiple nuclei and numerous apical microvilli. The nuclear chromatin distribution was uniform as follows: euchromatin was abundant; heterochromatin was distributed along the nuclear membrane. Mitochondria were usually round or oval, and ERs had developed in the cytoplasm. The BM of STs was continuous, uniform, and thin basal lamina, which was identical to the BM of fetal endothelium. The stroma was the connective tissue core of the chorionic villi between the BMs of STs and fetal endothelium.

In the GDM group, the ultrastructural characteristics of terminal villi that differed from those of the control group were showed in Figures 2(d) to 2(f). The following appearances were found in GDM placentas: the degenerative alterations of terminal villi, the thick BM of VSM and VSM itself, significantly reduced number of or even absent ST microvilli, swollen or even completely destroyed mitochondria, and ERs and STs interspersed with multiple vacuoles. In some areas of GDM placentas, microvilli were completely devoid.

On the TEM, structures of mitochondria, vesica, and endoplasmic reticulum (ER) were further observed. Figures 2(g) to 2(i) showed mitochondria, vesica, and ER in ST cytoplasm of control group. In the GDM group, massive swelling, even ridge deprivation, and architectural disruption of mitochondria were found (Figure 2(j)), and dilations of cisternae (Figure 2(k)) and ER (Figure 2(l)) were also found.

### 3.3. Quantitative Assay

Comparisons of semiquantitative parameters (as described in Table 2) of placental ultrastructure between GDM and control group were summarized in Table 3. There were significant differences in those structural characteristics of cytoplasmic vacuoles, stroma edema, mitochondria, and endoplasmic reticulum (*P* < 0.01).

**Figure 3:**
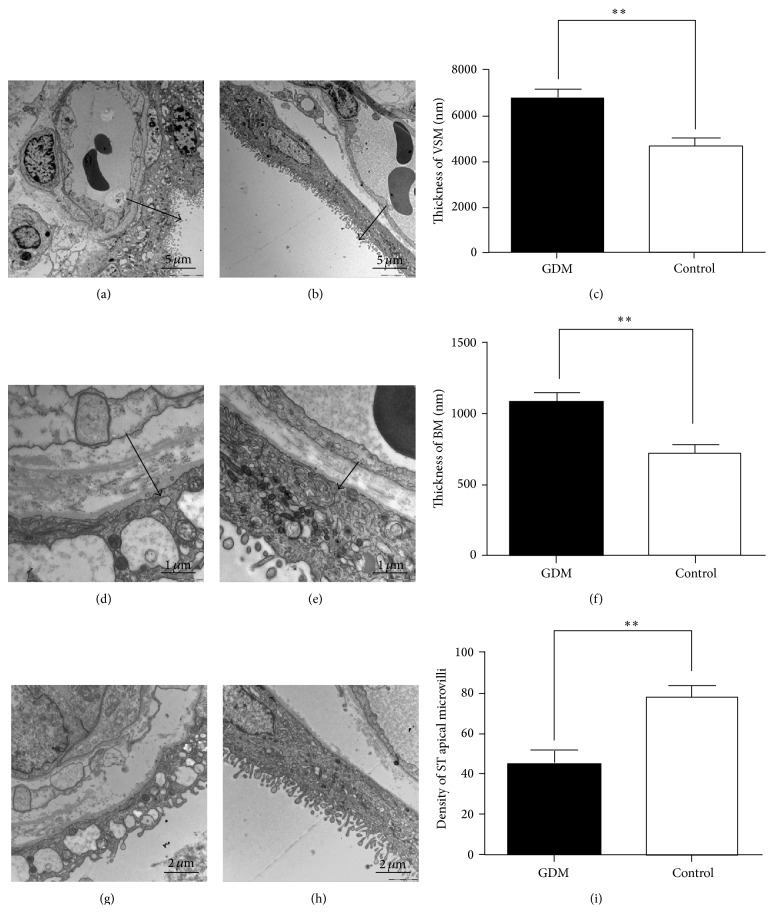
Comparison of semiquantitative parameters of placental ultrastructures between GDM and control group. (a–c) The terminal villi of GDM (a) and control (b) placenta. (c) Placental barrier thickness (arrow) of GDM group (6746.15 ± 1270.22 nm, *n* = 10) was significantly thicker than that of control group (4591.34 ± 1178.60 nm, *n* = 10; *P* < 0.05). Bar = 5 um. (d–f) The terminal villi of GDM (d) and control (e) placenta. (f) The BM of ST in GDM group (1077.49 ± 194.39 nm nm, *n* = 10) was thicker than that in control group (707.54 ± 256.56 nm, *n* = 10; *P* < 0.05). Bar = 1 um. (g–i) The density of ST apical microvilli in GDM (g) and control (h) placenta. (i) The density of ST apical microvilli in GDM group (44.36 ± 21.95 per 10 um, *n* = 10) was significantly lower than that in control group (77.13 ± 20.82 per 10 um, *n* = 10; *P* < 0.05) and even microvilli-free in some areas. Bar = 2 um.

The VSM and BM of ST were significantly thicker in the GDM group (6,746.15 ± 1,270.22 nm and 1,077.49 ± 194.39 nm, resp.; Figures 3(a) and 3(d)) than those in the control group (4,591.34 ± 1,178.60 nm and 707.54 ± 256.56, resp.; Figures 3(b) and 3(e)) (*P* < 0.05). The density of ST apical microvilli per unit surface area in the GDM group (44.36 ± 21.95 per 10 *μ*m) was significantly lower than that in the control group (77.13 ± 20.82 per 10 *μ*m) (*P* < 0.05), as showed in Figures 3(g) to 3(i).

## 4. Discussion

Hyperglycemia and hypoxia are two key factors in the pathophysiological process of GDM complications, and, in GDM, it is hyperglycemia that induces hypoxia and oxidative stress in placenta by several pathways, including leukostasis, vasoconstriction, and a proinflammatory situation [11–15]. Therefore, hyperglycemia in GDM is a proconstricting [11], procoagulatory [12], proinflammatory [13], proangiogenic [14], and propermeability [15] factor which affects vasculogenesis, angiogenesis and maturation of vascular system, and vascular dysfunction during critical periods of placental development [16]. Some significant changes in the placental barrier in GDM were found, including the thickened placental barrier, decreased density of ST apical microvilli, and increased ST vacuoles, all of which potentially inhibit transplacental transport and exchange. To our knowledge, the present study is the first to investigate systematically the ultrastructural changes in human term placentas derived from women with GDM.

The VSM of terminal villi is the most important structure of placental barrier, which determines the diffusion distance between maternal blood and fetal blood. Mayhew et al. found that those changes in the preeclampsia placenta were related to the fetal intrauterine growth restriction (IUGR), including the exchange surface areas, diffusion distances, and villous membrane diffusive conductance [17]. They found that the IUGR placentas showed the thick BM of ST and VSM itself, which changed the capacity of diffusional transfer across placenta. In addition, the thickening placental barrier is also found in the cases of women who have conceived using assisted reproductive technology [18], who smoke [19], or who have preeclampsia or eclampsia [20]. Vranes et al. observed that the loss of blood vessel elasticity in diabetes was possibly related to the collagen deposition in tunica media [21]. The decreased elasticity of vessel wall leads to vascular hardening and increased susceptibility to atherosclerosis [22], whereas, in the microcirculation, alteration in the BMs of arterioles leads to weakening and dilation of the capillary walls, with a tendency to rupture (microangiopathy) [23, 24]. These changes of the structure and function of villous capillaries in placenta would disrupt the environment of fetus development, such as hypoxia and epigenetic influences [25]. In this study, the thick VSM and BM of VSM were observed in GDM placenta. These changes may adversely affect the transport efficiency of placental vasculature, such as the decreasing transport of oxygen, nutrients, and waste.

The microvilli projecting from the apical portion of ST appear to be highly pleomorphic and show regional variations in distribution. The majority of fetal/maternal exchange occurs at the terminal branches of chorionic villi [26]. The apical microvillous density [27] and the surface area are related to the degree of trophoblastic maturation, and placental maturation is the most discriminative and by far the most important feature that needs to be assessed in diagnosis of the chronic hypoxia in utero [28]. Loss of microvilli and gross thinning of syncytium with distorted microvilli have been reported in the terminal villi of placentas from women with eclampsia [20]. The density of apical microvilli has been observed to be considerably reduced, and the occasional microvilli-free areas have been observed in IUGR and small-for-gestational-age fetuses [29]. These findings indicated the decreased exchange of gases, nutrients, and waste between maternal and fetal circulatory systems. Previous quantitative studies on the villi of placenta from women with well-controlled diabetes mellitus concluded that normal values were preserved by good glycaemic control regardless of diabetic grouping [30]. Therefore, stereological comparison of 3D spatial relationships involving villi and intervillous pores did not differ significantly in placentas from diabetic subjects with good glycaemic control [31]. Another study presented that uteroplacental blood flows were decreased in women who went on to develop preeclampsia, which indicated that poor glycemic control during pregnancy was associated with the development of preeclampsia [32]. In the present study, our results showed an association between microscopic and ultrastructural changes and the development of diabetes mellitus, which were conflicting with previous reported data. It may be explained by the fact that women in our studies have developed to adverse pregnancy outcome, such as preeclampsia. In the present study, a significantly decreased number of ST microvilli were observed in the GDM placentas. Some STs microvilli appeared to disappear completely. These changes of ultrastructure in GDM placenta would affect transplacental transfer, metabolism, and oxygen-diffusing.

It was also found that there were much more STs vacuolated in GDM placenta when compared with the control group. In the in vitro cultured model of placental villous, ST showed the cytoplasmic vacuolization and subsequent degeneration which was related to preeclampsia [33]. Hyperglycemia and hypoxia in GDM may enhance the lysosome/vacuole functions of ST, which results in the widespread cytoplasmic vacuolization and the altered transplacental metabolic exchange. Mitochondria and ERs are the most vulnerable organelles, which are susceptible to hyperglycemia and hypoxia [34–36]. Mitochondria are double-membrane organelles with multiple essential functions, such as cellular survival, energy metabolism, and intracellular ATP production by oxidative phosphorylation [35, 36]. ER synthesizes many secretary proteins and other factors, lipids, and membrane phospholipids, which participates in steroid synthesis in ST. The dilation and vacuolization of mitochondria and ER were most evident in the in vitro experiments and the in vivo samples of placental tissues from women with preeclampsia combined with IUGR [36]. In our study, we found massive swelling, ridge deprivation, and architectural disruption of the mitochondria and dilation of ER cisternae (Figure 2). The abnormal ultrastructures of mitochondria and ER could have impacts on metabolic functions and synthesis in ST.

In summary, our data showed the distinct alternations in the morphology and ultrastructure of GDM placentas which formed some compensatory mechanism to maintain homeostasis at the maternal-fetal interface in GDM. We assumed that those changes in the placenta and maternal-fetal interface are related to the pregnancy complications, adverse outcomes, and even fetal origin diseases. Moreover, because of the limited sample size, the present study is just the starting point in evaluating pregnancy outcome of GDM. Large-scale analyses and molecular studies are necessary to evaluate the short- and long-term effects of ultrastructural changes of GDM placenta on both offspring and mother.

## Supplementary Material

Supplemental Table 1: The detailed clinical information of two groups. Ten pregnant women with GDM (as GDM group) and ten normal pregnant women (as control group) were included in this study. The diagnosis of GDM is made when any of the following plasma glucose values are exceeded based on the American Diabetes Association (2011) [1]: fasting=5.1 mmol/l (92 mg/dl); 1 hour=10.0 mmol/l (180 mg/dl); 2 hours=8.5 mmol/l (153 mg/dl). Women with a history of pregestational diabetes and those with a non-singleton index pregnancy were excluded. After GDM was diagnosed, those GDM women were asked diet control to meet the satisfying range of fasting blood glucose (3.3 to 5.6 mmol/L). All placenta samples from them with the caesarean delivery, and there were not significant differences in the age of pregnant women and the gestational weeks at delivery between two groups. And there were not significant differences in the birth weight and the sexual ratio of babies between two groups.
Supplemental Table 2. The detailed clinical information of two groups. A 75-goral glucose tolerance test (OGTT) was performed with plasma glucose measurement fasting and at 1 and 2 h for women at 24–28 weeks of gestation. After GDM was diagnosed, those GDM women were asked diet control to meet the satisfying range of fasting blood glucose (3.3 to 5.6 mmol/L). Until delivery, eight GDM women (8/10) kept in the A1 class (fasting glucose less than 5.8 mmol/L, postprandial blood glucose less than 6.7 mmol/L) after good diet control (Supplemental Table 1). Two GDM women (2/10) were classed as A2 (fasting blood glucose higher than or equal to 5.8 mmol/L, postprandial blood glucose higher than or equal to 6.7 mmol/L) because of their poor diet control. The levels of fasting blood glucose and HbA1c in those GDM women with good diet control in the last weeks of gestation were kept in the range of 5.1 to 5.6 mmol/L. However, level of fasting blood glucose (6.1 and 11.3 mmol/L) and level of HbA1c (10.2 and 10.3 mmol/L) in two GDM women with poor diet control were higher than the satisfactory criteria. Women with normal pregnancies matched with GDM women for number of gestational weeks, maternal age and mode of delivery were recruited as control.

## Figures and Tables

**Table 1:** Clinical characteristics of women with GDM (*n* = 10) and control group (*n* = 10).

Characteristics	GDM group	Control group	*P* value
	(*n* = 10)	(*n* = 10)	
Maternal age (yr)	30.40 ± 4.90	28.50 ± 3.57	0.34
Weight (kg)	83.55 ± 12.03	73.20 ± 6.52	0.03
Nulliparous (*n*)	7	8	—
Gestational weeks at delivery	39.00 ± 0.90	39.41 ± 0.96	0.34
Mode of delivery	Caesarean	Caesarean	—
Birth weight (g)	3690 ± 640	3690 ± 390	1.0
Infant sex			
Male	5	4	—
Female	5	6	—

**Table 2:** Terminal villi were evaluated with respect to cytoplasmic vacuoles, pyknosis, and edema in the stroma, according to the semiquantitative score based on the degree of ultrastructural change.

Characteristics	0	1	2	3
Cytoplasmic vacuoles	None	Sporadic vacuoles	Moderate degenerative vacuolation	Distinct degenerative vacuolation
Pyknosis	None	Mild pyknosis	Moderate pyknosis	Obvious pyknosis
Stroma edema	None	Mild	Moderate	Massive
Mitochondria	None	Mildly swollen	Obviously swollen	Completely destroyed
Endoplasmic reticulum	None	Mildly swollen	Obviously swollen	Completely destroyed

**Table 3:** The semiquantitative analysis of placental ultrastructures in the GDM group and control group.

Characteristics	Semiquantitative score	GDM	Control	*P* value
		(*n* = 150)^#^	(*n* = 150)^#^	
Cytoplasmic vacuoles	0	12 (8%)	75 (50%)	0.000
	1	18 (12%)	30 (20%)	—
	2	42 (28%)	21 (14%)	—
	3	78 (52%)	24 (16%)	—

Pyknosis	0	22 (14.7%)	13 (8.7%)	0.106
	1	35 (23.3%)	73 (48.7%)	—
	2	71 (47.3%)	45 (30%)	—
	3	22 (14.7%)	19 (12.6%)	—

Stroma edema	0	27 (18%)	82 (54.7%)	0.000
	1	28 (18.7%)	35 (23.3%)	—
	2	36 (24%)	21 (14%)	—
	3	59 (39.3%)	12 (8%)	—

Mitochondria	0	11 (7.3%)	79 (52.7%)	0.000
	1	21 (14%)	48 (32%)	—
	2	42 (28%)	16 (10.7%)	—
	3	76 (50.7%)	7 (4.6%)	—

Endoplasmic reticulum	0	24 (16%)	88 (58.7%)	0.000
	1	31 (10.7%)	37 (24.7%)	—
	2	52 (34.7%)	21 (14%)	—
	3	43 (28.6%)	4 (2.6%)	—

^#^Fifteen fields of vision were randomly recorded and analyzed per placenta in order to minimize the individual differences.
