# Supplementary material for: Ultrastructure of Placenta of Gravidas with Gestational Diabetes Mellitus
Source: Obstet Gynecol Int. 2015 Aug 24;2015:283124. doi: 10.1155/2015/283124 (PMC4561319; doi:10.1155/2015/283124)
Supplement: Supplementary file 1 — Supplemental Table 1: The detailed clinical information of two groups. Ten pregnant women with GDM (as GDM group) and ten normal pregnant women (as control group) were included in this study. The diagnosis of GDM is made when any of the following plasma glucose values are exceeded based on the American Diabetes Association (2011) [1]: fasting=5.1 mmol/l (92 mg/dl); 1 hour=10.0 mmol/l (180 mg/dl); 2 hours=8.5 mmol/l (153 mg/dl). Women with a history of pregestational diabetes and those with a non-singleton index pregnancy were excluded. After GDM was diagnosed, those GDM women were asked diet control to meet the satisfying range of fasting blood glucose (3.3 to 5.6 mmol/L). All placenta samples from them with the caesarean delivery, and there were not significant differences in the age of pregnant women and the gestational weeks at delivery between two groups. And there were not significant differences in the birth weight and the sexual ratio of babies between two groups. Supplemental Table 2. The detailed clinical information of two groups. A 75-goral glucose tolerance test (OGTT) was performed with plasma glucose measurement fasting and at 1 and 2 h for women at 24–28 weeks of gestation. After GDM was diagnosed, those GDM women were asked diet control to meet the satisfying range of fasting blood glucose (3.3 to 5.6 mmol/L). Until delivery, eight GDM women (8/10) kept in the A1 class (fasting glucose less than 5.8 mmol/L, postprandial blood glucose less than 6.7 mmol/L) after good diet control (Supplemental Table 1). Two GDM women (2/10) were classed as A2 (fasting blood glucose higher than or equal to 5.8 mmol/L, postprandial blood glucose higher than or equal to 6.7 mmol/L) because of their poor diet control. The levels of fasting blood glucose and HbA1c in those GDM women with good diet control in the last weeks of gestation were kept in the range of 5.1 to 5.6 mmol/L. However, level of fasting blood glucose (6.1 and 11.3 mmol/L) and level of HbA1c (10.2 and 10. [file 283124.f1.pdf]

Supplemental Table 1. The detailed clinical information of two groups. Ten pregnant women with GDM (as GDM group) and ten normal pregnant women (as control group) were included in this study. The diagnosis of GDM is made when any of the following plasma glucose values are exceeded based on the American Diabetes Association (2011) [1]: fasting≥5.1 mmol/l (92 mg/dl); 1 hour≥10.0 mmol/l (180 mg/dl); 2 hours≥8.5 mmol/l (153 mg/dl). Women with a history of pregestational diabetes and those with a non-singleton index pregnancy were excluded. After GDM was diagnosed, those GDM women were asked diet control to meet the satisfying range of fasting blood glucose (3.3 to 5.6 mmol/L). All placenta samples from them with the caesarean delivery, and there were not significant differences in the age of pregnant women and the gestational weeks at delivery between two groups. And there were not significant differences in the birth weight and the sexual ratio of babies between two groups.

Cases	Age (yr)	Weight (kg)	Gravidity	Previous parity	Gestational wk at delivery	Mode of delivery	Sex of baby	Birth weight (g)
**GDM 1**	29	80	1	0	40.3	Caesarean	Male	3800
**GDM 2**	31	67.5	4	0	39.2	Caesarean	Female	5000

**GDM 3**	27	97	1	0	38.0	Caesarean	Male	2750
**GDM 4**	30	97	1	0	39.7	Caesarean	Male	3850
**GDM 5**	28	64	1	0	38.1	Caesarean	Female	3100
**GDM 6**	36	93	3	2	39.7	Caesarean	Female	3750
**GDM 7**	31	76	2	0	38.0	Caesarean	Male	3250
**GDM 8**	27	95	1	0	39.1	Caesarean	Female	4150
**GDM 9**	24	81	3	2	38.0	Caesarean	Male	3250
**GDM 10**	41	85	3	1	39.9	Caesarean	Female	4000
**Cont 1**	27	81	1	0	40.7	Caesarean	Female	3500
**Cont 2**	29	68	2	0	39.3	Caesarean	Female	4100
**Cont 3**	26	69	1	0	40.0	Caesarean	Male	3250
**Cont 4**	28	67.5	5	1	38.9	Caesarean	Female	3600
**Cont 5**	28	80	1	0	38.4	Caesarean	Male	3850
**Cont 6**	28	71	1	0	38.0	Caesarean	Male	4000

**Cont 7**	23	66	2	0	40.4	Caesarean	Female	3450
**Cont 8**	23	72	3	1	40.4	Caesarean	Female	4000
**Cont 9**	35	85	2	0	38.4	Caesarean	Female	3000
**Cont 10**	27	72.5	1	0	39.6	Caesarean	Male	4150

Supplemental Table 2. The detailed clinical information of two groups. A 75-goral glucose tolerance test (OGTT) was performed with plasma glucose measurement fasting and at 1 and 2 h for women at 24–28 weeks of gestation. After GDM was diagnosed, those GDM women were asked diet control to meet the satisfying range of fasting blood glucose (3.3 to 5.6 mmol/L). Until delivery, eight GDM women (8/10) kept in the A1 class (fasting glucose less than 5.8 mmol/L, postprandial blood glucose less than 6.7 mmol/L) after good diet control (Supplemental Table 1). Two GDM women (2/10) were classed as A2 (fasting blood glucose higher than or equal to 5.8 mmol/L, postprandial blood glucose higher than or equal to 6.7 mmol/L) because of their poor diet control. The levels of fasting blood glucose and HbA1c in those GDM women with good diet control in the last weeks of gestation were kept in the range of 5.1 to 5.6 mmol/L. However, level of fasting blood glucose (6.1 and 11.3 mmol/L) and level of HbA1c (10.2 and 10.3 mmol/L) in two GDM women with poor diet control were higher than the satisfactory criteria. Women with normal pregnancies matched with GDM women for number of gestational weeks, maternal age and mode of delivery were recruited as control.

**Cases**	**Fasting blood-glucose in the last week of delivery(mmol/L)**	**HbA1c in the last week of delivery(mmol/L)**	**complication**	**treatment protocol**
GDM1	5.2	5.6	-	Diet control

GDM2	6.1	10.2	hydramnios	-
GDM3	5.1	5.0	-	Diet control
GDM4	5.6	7.2	Pre-eclampsia	Diet control
GDM5	11.3	10.3	Pre-eclampsia	-
GDM6	5.5	6.2	-	Diet control
GDM7	5.6	5.8	-	Diet control
GDM8	5.2	7.1	Pre-eclampsia	Diet control
GDM9	5.6	7.3	Pre-eclampsia	Diet control
GDM10	5.4	5.6	-	Diet control
Cont1	4.1			
Cont2	4.3			
Cont3	4.1			
Cont4	4.3			
Cont5	4.2			
Cont6	3.8			
Cont7	3.9			
Cont8	4.3			
Cont9	3.6			
Cont10	3.1			
